# Cancer cell death induced by ferritins and the peculiar role of their labile iron pool

**DOI:** 10.18632/oncotarget.25416

**Published:** 2018-06-15

**Authors:** Juan Carlos Cutrin, Diego Alberti, Caterina Bernacchioni, Silvia Ciambellotti, Paola Turano, Claudio Luchinat, Simonetta Geninatti Crich, Silvio Aime

**Affiliations:** ^1^ University of Torino, Department of Molecular Biotechnology and Health Sciences, Torino, Italy; ^2^ Center for Magnetic Resonance, University of Florence, Florence, Italy; ^3^ IBB-CNR, Sede Secondaria c/o MBC, Torino, Italy

**Keywords:** ferritin, iron release, cancer therapy, HeLa cells, TFR1

## Abstract

Cellular uptake of human H-ferritin loaded with 50 or 350 iron ions results in significant cytotoxicity on HeLa cells at submicromolar concentrations. Conversely, Horse Spleen Ferritin, that can be considered a model of L-cages, as it contains only about 10% of H subunits, even when loaded with 1000 iron ions, is toxic only at >1 order of magnitude higher protein concentrations. We propose here that the different cytotoxicity of the two ferritin cages originates from the presence in H-ferritin of a pool of non-biomineralized iron ions bound at the ferroxidase catalytic sites of H-ferritin subunits. This iron pool is readily released during the endosomal-mediated H-ferritin internalization.

## INTRODUCTION

Ferritin is a protein highly conserved in living organisms, which stores iron as a bioavailable ferric-oxide mineral [1–3].

In animals, the 24-meric cage structure is assembled from two subunits, the heavy chain (H-chain) and light chain (L-chain), or a mix thereof. The two distinct subunits share a similar fold and 53% of sequence identity, but differ in their ability to handle iron. H-subunits contain a catalytic center (ferroxidase or oxidoreductase site) where iron(II) is oxidized to iron(III) by dioxygen on the ms time scale [4]. L-subunits lack the ferroxidase site; iron oxidation in L-type cages occurs on a time scale of the order of minutes and the protein mainly provides a shell to maintain the ferric oxide biomineral in a soluble form [5].

The demineralized form of L-type ferritin (apoferritin) has been extensively used for drug cellular delivery. Encapsulation of doxorubicin [6], curcumin [7], cisplatin [8] and desferrioxamine B [9] are only few examples. The selective ability of cells to internalize L- and H-cages through specific receptors has been demonstrated, namely SCARA5 (Scavenger receptor class A type 5) for L-rich cages and TfR1 (Transferrin receptor 1) for H-rich cages [10, 11].

Based on evidence attained from both epidemiological and molecular studies, new insights are linking the presence of excess iron and altered iron metabolism to cancer [12]. It is well established that many cancer cells reprogram iron metabolism in ways that result in net iron influx [13]. Iron starvation can impair cell development and growth, whereas excessive iron accumulation leads to cytotoxicity, resulting from an over-production of free radicals. Actually, it has been reported that certain types of tumor cells (principally those with c-myc and h-ras oncogenes expression) appear unable to deal properly with the excess iron, as a consequence of ferritin and ferroportin down-regulation [14, 15].

Herein we report our observations on cell death triggering, based on the delivery of iron to tumor cells, by the administration of iron-loaded ferritins.

## RESULTS

### Iron-loaded ferritins are toxic for HeLa cells

To compare the effects of H- and L-ferritin on tumor cells, we sought a tumor cell model that expresses both TfR1 and SCARA5 receptors, such as the HeLa cells. This choice allowed us to compare the behavior of the two proteins in the same cell line.

In literature, a plethora of nanocarriers encapsulating different species within the natural horse spleen cage is available, as recently reviewed in [16]. This cage has the advantage of being commercially available and can be considered a model of L-cages, as it contains only about 10% of H subunits [17]. Horse spleen nanocages will be referred to as HoS-ferritin, hereafter. They have been studied in their apo form (HoS-apoferritin), in its commercially available form loaded with an average of 1000 iron ions/cage (HoS-1000), as determined by ICP-MS, and in two forms with lower loading (namely HoS-50 and HoS-350) for comparison with H-ferritin (see below).

Recently human H-ferritin has been proposed [6] as a potentially superior carrier for anticancer drug delivery because, given its human origin, it should not activate inflammatory or immunological response. Efficient heterologous expression methods for human H-ferritin (≥100 mg/L of purified protein, in our hands) [4, 18] make it attractive also from the economical point of view. As for HoS-ferritin, H-ferritin can also be loaded with variable amounts of iron. The first two iron ions/subunit (48 iron ions per cage) localize in the ferroxidase site; addition of more iron ions initiates the biomineralization reaction [4]. Here, we investigated two H-ferritin cages differently loaded with iron, namely: i) one containing about 50 iron ions per cage (H-50, hereafter), which corresponds to the full saturation of the 24 ferroxidase sites; ii) the other, containing 350 iron ions per cage (H-350, hereafter), which corresponds to the full saturation of ferroxidase sites plus initial formation of the biomineral core as demonstrated in the TEM images (see Supplementary Figure 1). As expected the iron oxide core size, determined by TEM image analysis, was smaller in both H-350 (3.3 ± 0.7 nm) and HoS-350 (2.6 ± 0.2 nm) due to the lower iron content with respect HoS-1000 (4.4 ± 0.4 nm), whereas HoS-50 and H-50 didn't show any measurable core.

**Figure 1:**
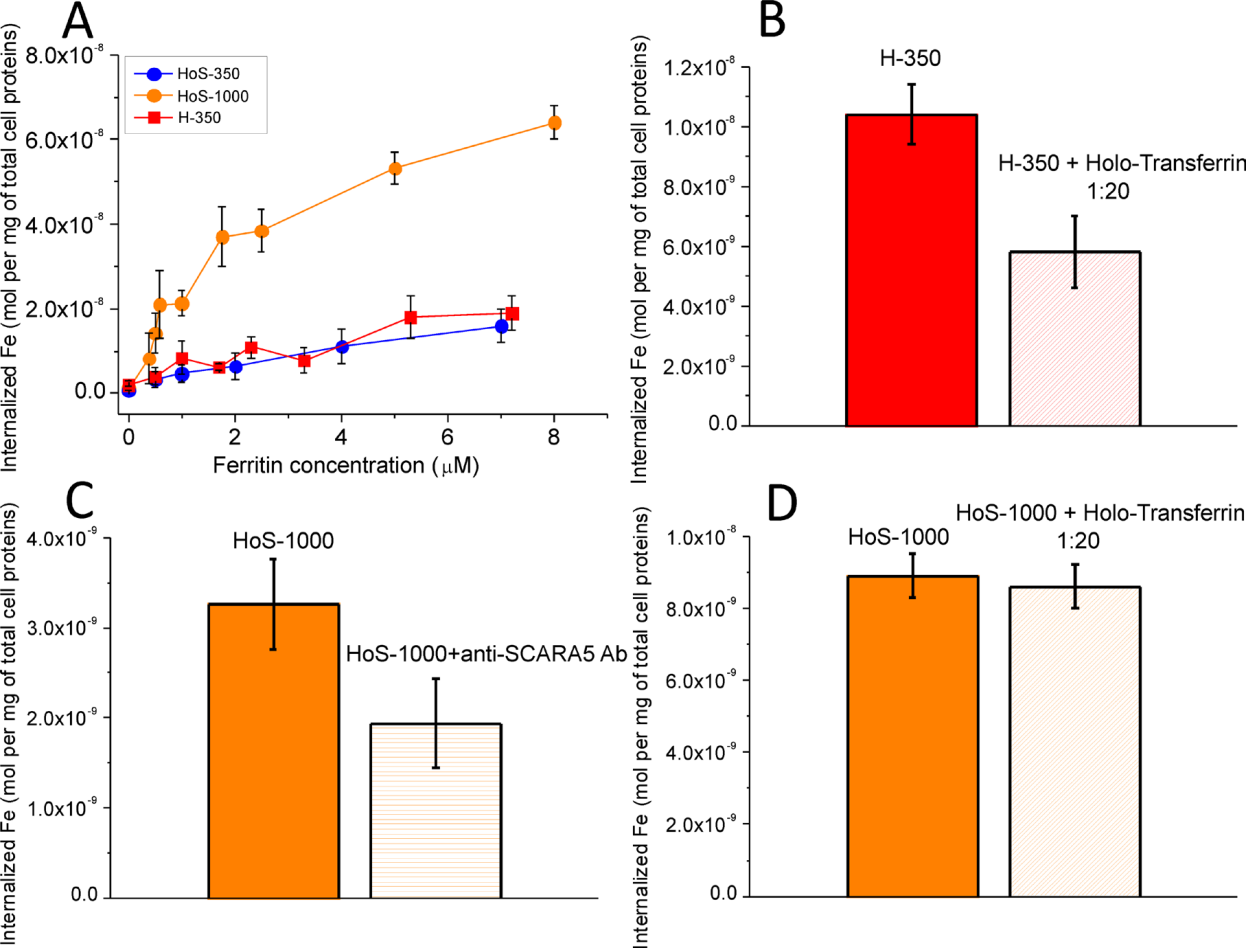
In cell studies: iron cell uptake (**A**) After 24 h incubation in the presence of increasing amounts of different ferritins, cell lysates were analyzed by ICP-MS to measure the amount of internalized iron, which was normalized to the total protein cellular content; (**B**, **C**, **D**) competition studies: iron uptake by Hela cells after 5 h incubation in the presence of (B) H-350 (1 μΜ) and HoS-1000 (D) with and without the addition of an excess of holo-transferrin (20 μM); (C) HoS-1000 0.5 μM with and without anti−SCARA5 antibody 5 mg/ml.

The amount of iron internalized by HeLa cells upon 24 h incubation in media containing variable concentrations of H- and HoS-ferritin was obtained by ICP-MS determination of the intracellular iron content (Figure 1A). The relative amount of internalized iron when using H-350, HoS-350 and HoS-1000 roughly correlates with the iron loading of the two carriers. The amount of internalized iron after the treatment with H-50 and HoS-50 in the 1–8 μM range was too low to be determined with sufficient accuracy; this finding is in line with what one could expect by scaling down the measured effect of H-350 and HoS-350 by a factor of 7 in iron concentration. To demonstrate that the uptake of the H-Ferritins involves TfR1, a competition assay was carried out treating Hela cells with an excess of Holo-transferrin. After 5 hours of cell incubation in the presence of H-350, the uptake of H-350 decreased by about 50 ± 8% when the concentration of Holo-transferrin added to the culture medium was 20 μM (Figure 1B). Since in the literature there are not competitors reported for SCARA5, the specific mediation of HoS-Ferritin uptake has been assessed by blocking experiments carried out with an anti-human SCARA5 antibody (anti-SCARA5 Ab, Figure 1C). The amount of iron internalized by cells treated with anti−SCARA5 Ab and incubated with HoS-1000 0.5 μM for 5h was 56 ± 21% than the amount internalized by control cells, thus confirming that SCARA5 mediates HoS-Ferritin uptake in Hela cells. Moreover, in order to assess whether the presence of 10% of H-chains in horse spleen ferritin used in this study can mediate its uptake through H-Ferritin receptors, a further competition study was carried out by incubating Hela cells for 5 hours with HoS-1000 (1 μM) in the presence of a 20-fold excess of Holo-transferrin. The Holo-transferrin excess caused a non significant reduction (3 ± 2%) of internalized iron thus ruling out the involvement of TfR1 receptors in the internalization of HoS-Ferritin (Figure 1D). The specificity of the ferritin targeting system was further confirmed by incubating H-350 and HoS-350 with an excess of H-Apo and HoS-Apo (Supplementary Information, Supplementary Figure 2). A significant decrease (>50%) of the internalized iron was observed only in the presence of the corresponding H-Apo or HoS-Apo forms. Vice versa no effect has been detected incubating H-350 and HoS-350 with an excess of HoS-Apo and H-350, respectively.

**Figure 2:**
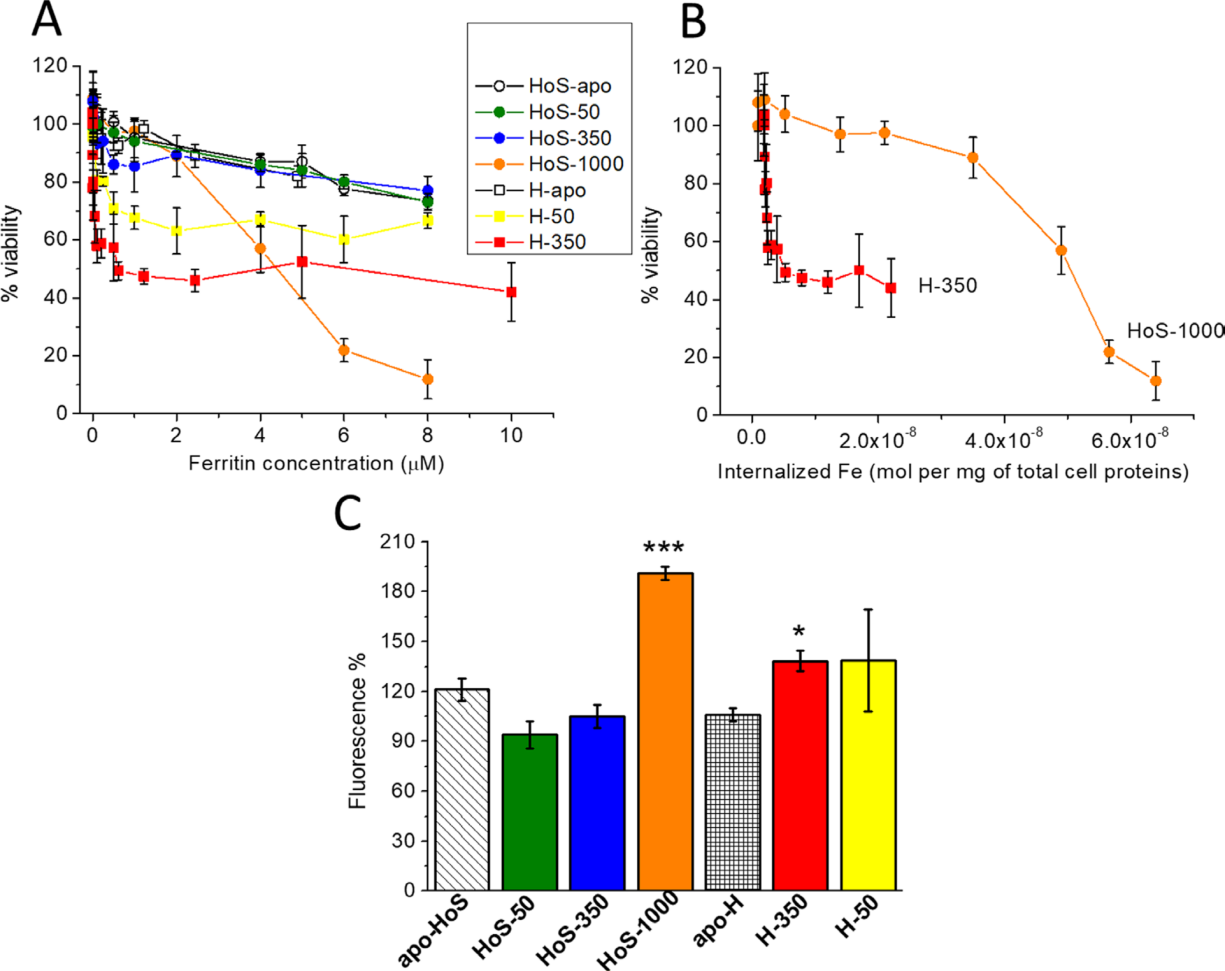
In cell studies: iron toxicity (**A**, **B**) HeLa cell viability measured by the MTT assay as a function of the concentration of the various ferritin forms (A) and as a function of internalized iron (B). Cell viability is expressed as percentage relative to untreated cells. Data are presented as mean and SD of at least three independent experiments. (**C**) ROS generation measured by DCF-DA fluorescence emission (at 529 nm) after the incubation, for 24 h, of HeLa cells with 5 μM different form ferritins. In all panels, error bars indicate the SD of the data. (Student's *t* test. HoS-1000 vs HoS−Apo: *P* = 0.0002; H-350 vs apo-H: *P* = 0.03; H-50 vs apo-H: *P* = 0.24; ^*^P < 0.05, ^***^P < 0.001). In panels A, B and C, HoS-apoferritin (HoS−Apo) and H-apoferritin (H−Apo) are represented as white circles and squares, respectively; HoS-1000 as orange circles, HoS-350 as blue circles, HoS-50 as green circles, H-350 as red squares and H-50 as yellow squares. In panel D, the bars are labelled and depicted with the same color code used in A, B and C.

Cell viability was determined by the MTT assay after treatment of HeLa cells for 24 h with apo and iron-loaded H- and HoS-ferritins. While both apo forms show no appreciable toxicity, a clear effect is induced by the iron-loaded cages (Figure 2A). Strikingly, the profiles of cellular cytotoxicity induced by the H- and L-cages are very different. The effect of HoS-1000 is almost linearly dependent on the protein concentration whereas the less loaded HoS-350 and HoS-50 showed a toxicity not significantly different from the apo form. On the contrary, treatment with either H-50 or H-350 causes an abrupt decrease in cellular viability, which levels off at about 50% viability for H-350 concentrations ≥1 μM ferritin. The attainment of a plateau value in cell viability for both iron-loaded H-ferritin nanocages can be interpreted in terms of iron-dependent downregulation of the TfR1 receptor expression; a well-known process in the case of incubation of HeLa with an excess of holo-transferrin or Fe-citrate [19, 20]. A flow cytometric analysis of the expression of TfR1 (Supplementary Figure 3) showed that the relative expression of this receptor with respect to untreated Hela cells decreases to 58 ± 5% for cells treated with 1 μM H-350 and to 75 ± 6% with 1 μM H-50, suggesting that also iron-loaded H-ferritins activate a downregulation mechanism. From the results shown in Figure 2A (lack of saturation effects in cytotoxicity when using HoS-1000), one may rule out the occurrence of an analogous iron-dependent SCARA5 down regulation response. In spite of the larger intracellular iron internalization induced by HoS-1000 with respect to H-350 or H-50, the latter two are much more toxic at lower protein concentrations. As illustrated in Figure 2B, for a given iron concentration, H-350 causes significantly larger reduction in cell viability, suggesting a different mechanism of action for H- and L-cages. Noteworthy, the comparison in the cytotoxic effect exerted by treatment with H-50 and H-350 is not proportional to their cage iron content. This result suggests that the nature of the iron ions within the cage (ferroxidase-bound or biomineralized) might play a role. In principle, one may surmise that ferroxidase-bound iron could be associated to the generation of toxic ROS. This aspect is further investigated in the following sections.

Cytoplasmic ROS production was evaluated by measuring 2,7-dichlorofluorescein diacetate (DCF-DA) emission; its fluorescence intensity directly correlates with the steady-state concentration of intracellular ROS. The treatment with 5 μM iron-loaded ferritins causes a modest increase in ROS production with respect to untreated cells and to cells treated with the apoferritins (Figure 2C). ROS generation by H-350 is only modestly higher than that of H-apo, while H-50 is indistinguishable from the other two, within the error. HoS-1000 induces a ROS production which is significantly higher than HoS−Apo, but still less than a factor of 2 whereas HoS-350 and HoS-50 are not significantly different. In conclusion, cytoplasmatic ROS production does not account for the striking differences in cell viability between H and L of Figure 2A. Consistently, at protein concentrations lower than 5 μM, where the H-350 and H-50 are heavily toxic (Figure 2A), no measurable ROS production is observed.

Moreover, in order to discriminate between an anti-proliferative or toxic- effect of ferritins, Hela cells have been incubated in the presence of H−50 (0.5 μM) or H−350 (0.5 μM) or L−1000 (3 μM). This treatment caused the death of about 30–40% of cells. Then the proliferation rates of the survived cells have been assessed over a period of 5 days. No significant difference has been noted between these cells and the untreated control cells (see Supplementary Figure 4). This finding led us to exclude that the reduced metabolism measured with the MTT assay is related to the occurrence of a reduced proliferation rate.

### The role of pH and endogenous reducing agents on iron release

**Figure 3:**
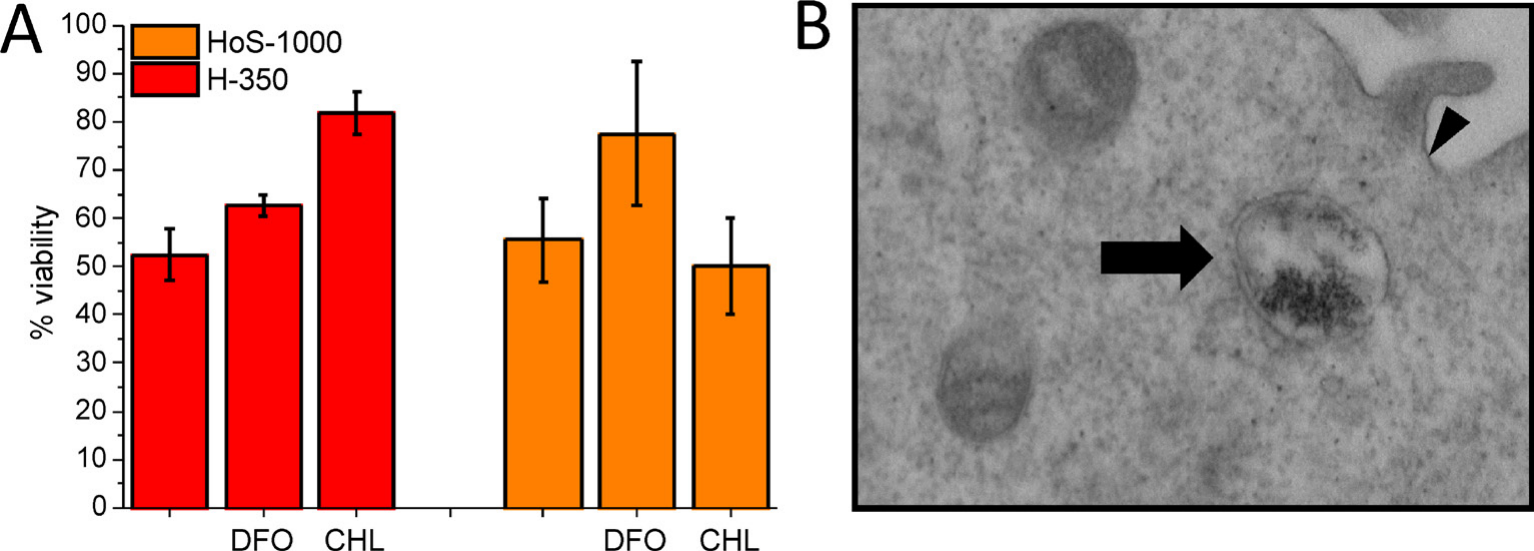
Iron release along the endosome/lysosome import system (**A**) HeLa cell viability measured by the MTT assay after 24 of incubation with H-350 (0.5 μM) or HoS-1000 (2.5 μM) in the absence and in the presence of DFO mesylate (150 μM) and Chloroquine (100 μM) ^**^P < 0.01, Student's *t*-test). (**B**). TEM pictures of Hela cells treated with HoS-1000. Arrows indicate representative endosomes. Hemosiderin bodies are observed only upon incubation with HoS-ferritin. The arrowhead signs correspond to the plasma membrane (×30.000, lead citrate and uranyl acetate stained).

As reported [10], TfR1-imported H-ferritin is initially localized in endosomes and then (in about 30 min) is distributed in both endosome and lysosomes. The cytotoxic effect of H-350 in the MTT assay carried out in the presence of chloroquine (CHL), an agent that limits endosomal acidification, resulted significantly reduced (*P* = 0.0076; Figure 3A). The effect is much smaller in the presence of DFO mesylate, a lysosomal iron chelator. The opposite behavior is observed for HoS-1000 (Figure 3A). Degradation of HoS-cages encapsulating massive biomineral particles (1000 iron/cage) gives rise to observable hemosiderin bodies [21] (Figure 3B). Accordingly, confocal microscopy studies (see Supplementary Figure 5) showed that HoS-ferritin was completely compartmentalized in lysosomes, whereas H-ferritin is only in part inside the lysosomal compartment. Using TfR1, H-ferritin was internalized by the receptor-mediated mechanism, only in part undergoing lysosomal degradation. Despite the much lower amount of delivered iron, H-ferritin appears definitively more toxic.

**Figure 4:**
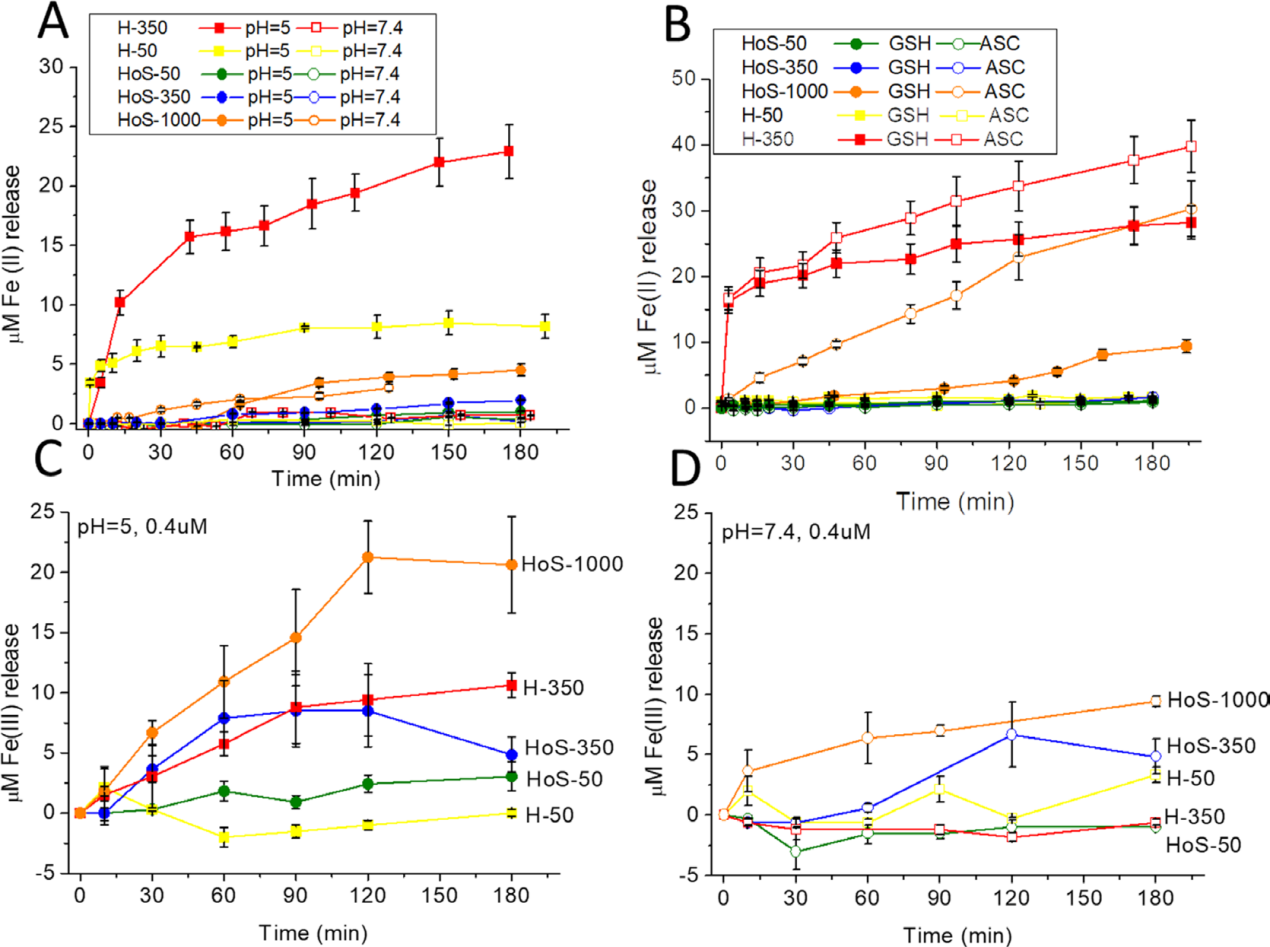
(**A**, **B**) Mobilization of iron(II) from 0.4 μM H-50, H-350, HoS-50. HoS-350 and HoS-1000 as a function of pH (A) and (B) in the presence of reducing agents (GSH 0.25 mM, pH = 7; ascorbic acid 0.25 mM, pH = 7). Samples were incubated with bipyridine (1 mM) under aerobic conditions. The amounts of iron(II) released by H-50 and H-350 account for a total of 21 iron/cage and 57 iron/cage, respectively. (**C**, **D**) Mobilization of iron(III) from 0.4 μM H-50, H-350, HoS-50. HoS-350 and HoS-1000 at pH = 5 (C) and pH = 7.4 (D). Samples were incubated with Xylenol Orange (0.25 mM) under aerobic conditions. T = 0 corresponds to the addition of ferritin to the solution media.

Mature endosomes are characterized by a low pH value (4.9–6.0) [22, 23]. At pH 5, only H-ferritin spontaneously releases iron(II), whereas at neutral pH the presence of reducing agents is required (Figure 4A). HoS-1000 is able to release iron(II) only in the presence of reducing agents (Figure 4B). The amounts of iron(II) released by H-50 and H-350 account for a total of 21 iron/cage and 57 iron/cage, respectively. The observed pH dependence of iron release *in vitro* suggests that in cells H-ferritin can release its metal load at the level of late endosomes, but HoS-1000 does not. In lysosomes, pH drops down to about 4.5; the harsher conditions combined with the possible degradation operated by lysosomal hydrolases [23] may cause cage disassembly. Moreover, iron(III) release has been determined using the orange xylenol based assay [24]. Xylenol orange has long been known as a good chelator for the quantitative determination of a wide range of cations, including iron. It binds Fe^3+^, but not Fe^2+^, at acid pH to give a colored complex [24] and the reaction is not sensitive to oxygen. Figure 4C and 4D show that HoS-1000 releases iron(III) at neutral and, in particular, at acid pH more than H-350 and H-50, whereas the iron(III) released from HoS-350 at acid pH is similar to H-350. While iron(III) release in the presence of xylenol orange seems to be proportional to the biomineral content, independently of the cage type, the release of iron(II) in the presence of 2,2′-bipyridine is selectively observed for H-ferritin. Organisms and cells keep this ferric iron in solution by secreting chelating agents able to bind Fe(III) more tightly than OH− and thus preventing the oxo-bridging of Fe(III) species that leads to its precipitation [25]. However, by stabilizing this Fe(III) state, the chelating agents set the Fe(III) reduction potential at a low value that prevents its participation as oxidant in reaction with a biologic reducing agent. This ‘stable’ ferric iron is inert in regard to the Fe(III) reduction reaction associated with normal iron metabolism.

### Relaxometric characterization

**Figure 5:**
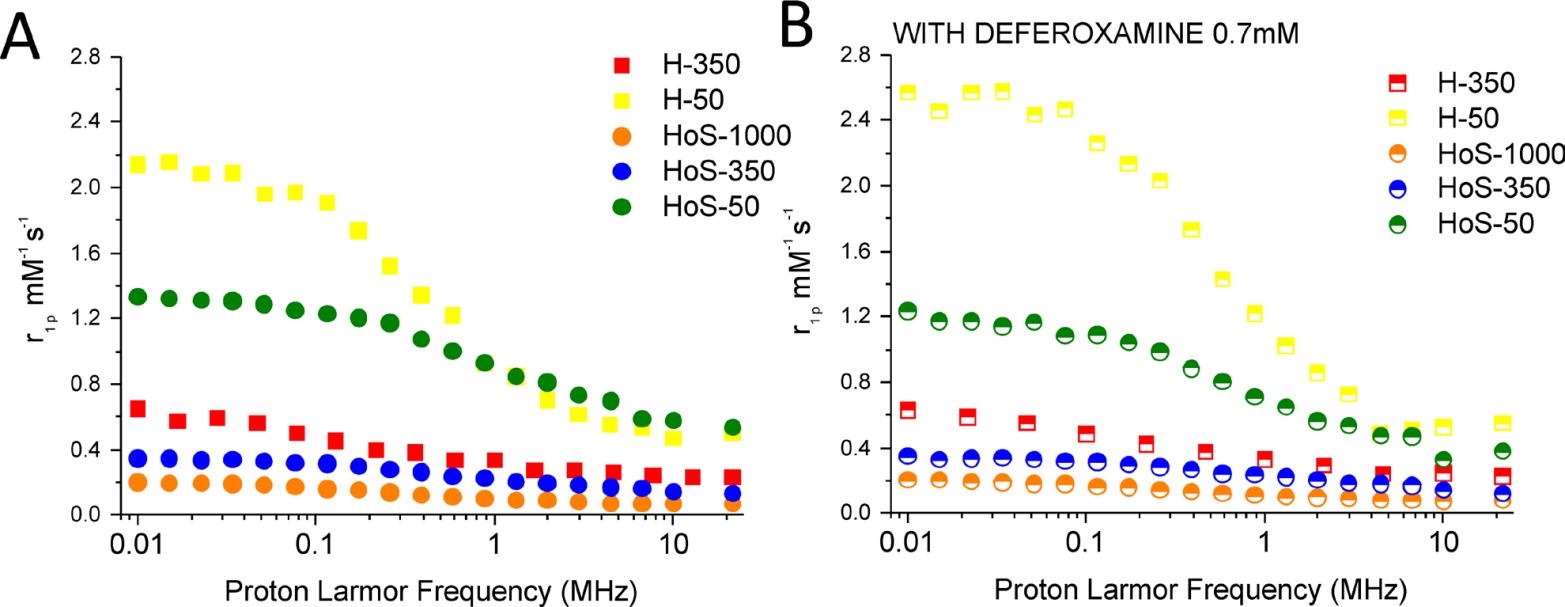
Ferritins as relaxation agents 1/T_1_ NMRD profiles (pH 7.4, 25° C) of H-50, H-350, HoS−50, HoS−350 and HoS-1000 (10 μM in protein). The profiles were recorded without (**A**) and with the ferritins treatment with DFO 0.7 mM (15’ at 25° C) (**B**), to avoid any possible contribution from spurious surface iron ions. The relaxivity values (r_1p_ mM^-1^s^-1^) were normalized to the iron concentration.

Horse spleen ferritin has been proposed as a carrier for MRI contrast agents [26, 27]; here, we investigate the relaxometric properties of ferritins with different iron loadings. 1/T_1_ NMRD profiles (Figure 5A) were recorded for H-50, H-350, HoS−50, HoS−350 and HoS-1000. The profiles are only marginally affected by addition of deferoxamine (DFO) that it is expected to bind only the iron fraction possibly present on the external surface of the protein (Figure 5B). The profiles indicate a negligible contribution from externally coordinated iron ions. The higher millimolar relaxivities at any field shown by H-50 and, to a lower extent by HoS-50, suggest a paramagnetic contribution from non-biomineralized iron (Figure 5). This contribution is less pronounced in H-350 and almost absent in HoS-1000. As reported [28], the differences in H/L ratio in ferritin cages determine different morphologies of the mineral core and this fact might influence the relaxivity profiles.

## DISCUSSION

### A structural biology view of different toxicity of H and L chains

Recent X-ray crystallography studies have demonstrated that the ferroxidase site of human H-ferritin accommodates two iron ions, named Fe1 and Fe2, that are bound to few protein amino acid side chains: a bridging carboxylate group is provided by Glu62; Fe1 is then bound to His65 and monodentate Glu27; Fe2 to bidentate Glu107; the coordination sphere of each iron ion is completed by water molecules. Nearby, there are two other additional binding sites involving the side chains of His57, Glu58 and Glu62, where hydrated iron ions are bound with low occupancy. The emerging picture is that of a cage that uptakes ferrous hexa-aqua ions through the C3 channels and delivers them (as progressively de-hydrated ions) towards the ferroxidase site under the effect of attractive electrostatic gradient created by carboxylate residues [29, 30]. Given the low number of protein residues involved in the binding of the iron at the accessory binding sites and in the ferroxidase site, it is reasonable to propose that they could be more readily released and react. The Fe1 and Fe2 sites remain always populated during subsequent reaction turnover, while the biomineral grows. Their iron release upon lowering pH can be explained in terms of protonation of the acidic residues involved in their binding. In contrast, in human L-ferritin and horse spleen ferritin, some of us have demonstrated that the iron ions that enter the C3 channels and react to form the biomineral precursors at the nucleation sites are blocked within (μ^3^-oxo)tris[(μ^2^-peroxo)(μ^2^-glutamato-κ*O*:κ*O*’)](glutamato-κ*O*)(diaquo)triiron(III) clusters. So they are probably more stable than iron ions in the ferroxidase site and lose their importance when the biomineral grows [31]. Furthermore, we can hypothesize an additional toxic effect due to the direct reaction of H_2_O_2_ generating OH. inside the protein cavity. In fact, as reported by Zhao and coworkers [32], H_2_O_2_ is able to react with the ferroxidase-bound iron(III), showing a stronger reactivity with the μ-oxo diferric complex than with the Fe(III) core which is more thermodynamically stable (in particular in the case of L-ferritin). The reactivity is inversely proportional to the size of the Fe(III) core.

### Therefore, the higher H-ferritin toxicity originates from its peculiar iron cargo as well by its pathway of interaction with the ELC

The characteristic behavior observed for H-ferritin, which shows toxicity effects that are not proportional to the iron-load, suggests a different behavior for the biomineralized caged iron and the “mobile” iron pool constituted by those metal ions that are at the relatively weakly binding ferroxidase sites and low-populated accessory binding sites in the catalytic center [4, 29, 33]. Indeed, the observed effects can be accounted for as originating by a pool of about 50 iron/cage over a total of 350/cage. Moreover the reduced acidification capacity of the ELC obtained by the treatment with CHL significantly reduces the H- but not HoS-ferritin induced cytotoxicity (Figure 3A). This divergent effect could be explained by the pH-dependent H-ferritin release of iron at the level of late endosomes, i.e. the neutralization of endosomal pH induced by CHL increases the iron retention by H-ferritin. The not-yet biomineralized iron could be released more easily upon acidification (Figure 4A) from the protein sites in the ferroxidase site, where the coordination sites are mainly provided by carboxylate and imidazole side chains. The redox activity, as measured by cytoplasmatic ROS generation, is only modestly increased from the apo- to the iron-loaded proteins and the increase is actually more modest for H than for L. We conclude that iron toxicity is only marginally dependent on ROS generation.

### The apparent H paradox

Our present findings rise an obvious question. Why a human protein is toxic for human cells? Here we have to consider a few key aspects: i) Endogenous ferritin is a cytoplasmic protein; under the cytosolic pH its cargo is stable and is not readily released [34]. ii) The natural cytoplasmic ferritin never undergoes endosomal-mediated import with consequent iron release induced by the low pH in endosome. iii) Only ferritin that could be externally administered via the blood stream can be internalized via TfR-1 receptors leading to endosomal internalization.

The expression of TfR1 on tumor cells is accounted for in terms of the needs for the fast growing tumor cells to import sufficient iron for the set-up of fundamental biological functions. However, when H-ferritin is involved in place of transferrin, the iron uptake may overcome the physiological needs and the toxic role of redox iron center come to prevail. The observed behavior may be accounted for in terms of the set-up of a spontaneous cell death process induced by iron-loaded H-ferritin taken up through TfR1 receptors.

In summary, the toxicity of iron-loaded H-ferritin is due to two main factors: the presence of a mobile iron pool, which has no counterpart in HoS-ferritins, and the lability of this pool at the low pH encountered during the endosomal import. On this basis, the exploitation of H-ferritins pro-toxic properties may open new horizons in the field of novel cancer therapies based on an iron dependent-mechanism that leads to cell death.

## MATERIALS AND METHODS

Apoferritin and iron-loaded ferritin from equine spleen (reported to contain > 90% of L subunits), Thiazolyl Blue Tetrazolium Bromide, DCF-DA, deferoxamine mesylate, chloroquine and all other chemicals were purchased from Sigma-Aldrich (St Louis, MO). The apo- and iron-loaded ferritin samples were used without any further purification and, given their composition, are hereafter named HoS−Apo and HoS−1000. The number of Fe/cage in iron-loaded HoS-ferritin was measured by ICP-MS.

### H-ferritin production/purification

The gene-coding sequence for human H-ferritin was custom-synthesized (GenScript, USA) and subcloned into pET-9a expression vector using NdeI and BamHI restriction sites. The expression plasmid was introduced by thermal shock into *E. coli* strain BL21 (DE3) pLysS cells. Transformants were selected in LB agar supplemented with 50 mg L^-1^ kanamycin. Cells were grown at 37**°** C until A_600 nm_ reached 0.6−0.8, and subsequently induced with isopropyl 1-thio-β-D-galactopyranoside (IPTG, 1 mM final concentration) for 4 h. Human H-ferritin was purified from the harvested cells, as described previously [4, 18]. Briefly, cells were broken by sonication and the cell free extract obtained after centrifugation (40 min, 40,000 rpm, 4° C) was incubated for 15 min at 65° C as the first purification step. After removal of the aggregated proteins (15 min, 40,000 rpm, 4° C), the supernatant solution was dialyzed against 20 mM Tris-HCl pH 7.5; applied to a Q-Sepharose column in the same buffer and eluted with a linear 0−1 M NaCl gradient in 20 mM Tris-HCl pH 7.5. Fractions containing ferritin, identified by Coomassie staining of SDS-PAGE gels, were combined and further purified by size exclusion chromatography using a Superdex 200 16/60 column. (GE Healthcare Life Sciences) Iron and other metal ions present in the purified ferritin were removed by four dialysis steps overday and at 4° C overnight, each using 4 L 20 mM Tris-HCl, 2.5 mM EDTA, 10 mL ammonium thioglycolate pH 7.5 to reduce and chelate the iron, followed by four dialysis steps at room temperature each using 4 L 20 mM Tris-HCl, pH 7.5.

### Preparation of iron-loaded H-ferritin and HoS−ferritin

Iron loading into human recombinant H-apoferritin homopolymer (100% H chain) and HoS−Apo from equine spleen was carried out by adding a FeSO_4_ solution (200 mM in 1 mM HCl at protein/Fe ratio of 1:500 and 30 mM in 1 mM HCl at protein/Fe ratio of 1:75) to a 4.5 μM protein solution dissolved in 100 mM MOPS, 100 mM NaCl pH 7 buffer. After the addition of the iron salt, the sample was vigorously vortexed for 5 sec, then left at 25° C for 2 hours and at 4° C overnight. The iron-loaded H-ferritin and HoS−ferritin were then purified by two cycle of dialysis at 4° C using 1 L of 4 mM Hepes, 150 mM NaCl pH 7.4 as external buffer. Final iron concentration was measured by ICP-MS. Samples of H-ferritin and HoS−ferritin were loaded with about 350 (H-350 and HoS−350) and 50 (H-50 and HoS−50) Fe/cage. The protein concentration was measured by the Bradford assay using Bovine Serum Albumin (BSA) as a standard.

### ICP-MS

Iron concentration was determined by using inductively coupled plasma mass spectrometry (ICP-MS; element-2; Thermo-Finnigan, Rodano (MI), Italy). Sample digestion was performed with concentrated HNO_3_ (70%, 1 mL) under microwave heating at 160° C for 8 minutes (Milestone MicroSYNTH Microwave labstation).

### NMRD profiles

The 1/T_1_ nuclear magnetic relaxation dispersion profiles (NMRD) of water protons were measured over a continuum of magnetic field strengths from 0.00024 to 0.5 T (corresponding to 0.01–20 MHz proton Larmor frequency) on a Fast Field Cycling relaxometer (Stelar Spinmaster FFC 2000 relaxometer) equipped with a resistive low inductance air cored silver solenoid [35]. The relaxometer operates under complete computer control with an absolute uncertainty in the 1/T_1_ values of ± 1%. The typical field sequences used were the NP sequence between 20 and 8 MHz and the PP sequence between 8 and 0.01 MHz. The observation field was set at 16 MHz. T_1_ was determined by the saturation recovery method. 16 values of delay (t) between pulses were used. The number of averaged experiments was 2.

### HeLa cell line

Cervical carcinoma (HeLa) cell line was obtained from the American Type Culture Corporation. They were cultured in RPMI 1640 medium (Lonza) containing 10% (v/v) fetal bovine serum (FBS), 2 mM glutamine, 100 U/ml penicillin, and 100 U/ml streptomycin. Cells were incubated at 37° C in a humidified atmosphere of 5% CO_2_. The cell line was routinely tested to make sure of the absence of mycoplasma contamination (MycoAlert™ PLUS Mycoplasma Detection Kit, Lonza).

### Uptake experiments

For uptake experiments, HeLa cells were seeded at a density of 2.5 × 10^5^ cells in a 25 cm^3^ culture flask and placed in a wet (37° C) 5% CO_2_ air atmosphere incubator. At 24 h post seeding, cells were incubated with increasing concentrations of the above-mentioned H−350, HoS−350 or HoS−1000. After 24 h of incubation, cells were washed three times with 10 mL ice-cold PBS, detached with trypsin/EDTA and sonicated for 30″ at 30% power in ice. The Fe content was determined by ICP-MS. The protein concentration (proportional to the cell number) was determined from cell lysates by the Bradford assay, using BSA as a standard.

### Proliferation assay

For proliferation assay, Hela cells were seeded at a density of 4 × 10^4^ cells in a 3.5 cm diameter petri dishes and placed in a wet (37° C) 5% CO_2_ air atmosphere incubator. At 24 h post seeding, four dishes were incubated with H−50 (0.5 μM) or H−350 (0.5 μM) or HoS−1000 (3 μM) for 24 h. Untreated cells were used as a control. At the end of the incubation, they were washed three times with 10 mL ice-cold PBS and only a dish for each incubated ferritins, detached with trypsin/EDTA, sonicated and transferred into falcon tubes. Then, cells were sonicated and the protein concentration (proportional to the cell number) from cell lysates was determined by the Bradford method. The remaining dishes were leaved in cell incubator at 37° C and 5% CO_2,_ detached with trypsin/EDTA at day 2,3 and 5 post incubation and their protein concentration was determined.

### Competition assays

To demonstrate that the uptake of H−350 involves TfR1 and in order to assess whether the presence of 10% of H-chains in HoS-ferritin can mediate its uptake through H-Ferritin receptors competition assays was carried out. Hela cells were seeded at a density of 2.5 × 10^5^ in a 6 cm diameter petri dishes and placed in a wet (37° C) 5% CO_2_ air atmosphere incubator. At 24 h post seeding, H−350 (1 μM) and HoS−1000 (1 μM) were incubated in the presence of Holo transferrin human (Sigma) 20 μM for 5 hours. Moreover, to demonstrate that the uptake of HoS−Ferritin involves SCARA5 receptors, 24 h post seeding, Hela cells were incubated with HoS-1000 (0.5 μM) for 5 h in the presence of 5 μg/mL anti−SCARA5 antibody (abcam ab118894) blocking the receptor. The competitions were evaluated by measuring Fe content by ICP−MS of cell lysates with respect to H−350 or HoS−1000 incubated in the same condition without the presence of competitor.

### FACS analysis of Transferrin Receptor expression

In order to perform FACS (Fluorescence Activated Cell Sorting) analysis, 2 × 10^5^ Hela cells were seeded in 3.5 cm of diameter culture dishes. The day after, cells were treated for 24 h with 0.34 mM Fe(III) citrate (Sigma Aldrich), 20 μM human Holo−transferrin (Sigma Aldrich) or 1 μM H−50 or 1 μM H-350. For the experiment, cells were grown in RPMI medium (Lonza). At the end of the incubation, the medium was removed and the cells were washed three times with PBS. Then, they were treated with a non-enzymatic dissociation solution (Sigma Aldrich) for 15 min at 37° C, transferred in 15 ml falcon tubes and counted in PBS. Cell number was determined using a cell sorting chamber (Burker-Turk chamber). The cells were divided in falcon tubes containing 1 × 10^6^ cells for each one.

All samples were centrifuged (1100 rpm for 5’), the PBS was removed, and the cells were further washed with PBS and centrifuged (1100 rpm for 5’). All the samples were incubated in 10% FBS (0.5 ml) for 30’ at 4° C, spun down (1100 rpm for 5’) and washed twice with PBS (3 ml). The PE conjugated antibody (mAb anti human CD71, BD Pharmingen) selective for TfR1 was added to the samples previously described (20 μl in 100 ul of 0.1% BSA/PBS) and to a non-treated sample of Hela cells. As a negative control, a mouse isotype control antibody PE-conjugated (Miltenyi Biotec) (5 ul in 100 μl of 0.1% BSA/PBS) was incubated to a non-treated sample of Hela cells. The incubation was performed at 4° C for 40’. All samples were washed with PBS (2 × 3 ml) and centrifuged (1100 rpm for 5’). Finally, cells were diluted in PBS (250 μl) and evaluated for their PE fluorescence on a flow cytometer (Becton Dickinson, FACS Calibur). Their PE fluorescence was analyzed using the CELLQUEST PRO program: the mean fluorescence intensity of the treated samples has been calculated as a percentage with respect to the non-treated Hela cells.

### MTT assay

MTT assay is based on the tetrazolium salts reduction to formazan by mitochondrial succinate dehydrogenase (SDH), which is quantified spectrophotometrically. 5 × 10^3^ Hela cells were seeded in a 96-well microtiter plate at 37° C and 5% CO_2_ air atmosphere. 24 h later, the cells were incubated for 24 h at 37° C and 5% CO_2_ with increasing concentration of H-50, H−350, HoS-50, HoS-350 and HoS−1000 and H- and HoS-apoferritin. Furthermore, H-350 (0.5 μM) and HoS-1000 (2.5 μM) were also incubated in absence or in the presence of DFO mesylate (150 μM) or chloroquine (100 μM) for 24 h. After this time, the medium was removed and 100 μl of Thiazolyl Blue Tetrazolium Bromide dissolved in medium at the concentration of 0.45 mg/mL was added into each well and the plate was incubated for 4 h at 37° C and 5% CO_2_. Then, the medium was removed, 150 μl of DMSO were added into each well to solubilize the formazan salt crystals produced by the metabolism of live cells and the microplate was incubated at room temperature for 30 minutes. Finally, absorbance was read at 570 nm with iMark microplate reader (Biorad). Cell viability was reported as percentage of death cells observed in treated samples relative to that observed in control cells. The experiment was performed in triplicate and the data were graphically presented as mean ± SD.

### Iron(II) mobilization from H- and HoS- ferritin

Iron loaded H- and HoS- ferritin (0.4 μM) have been incubated up to 3 h at 25° C in different buffers (Hepes/NaCl pH 7.4; 0.1 M acetate buffer pH 5) in the presence of 1 mM 2,2′-bipyridine under aerobic conditions. Incubations of proteins in Hepes/NaCl buffer at pH = 7.4 have been repeated in the presence of glutathione (0.25 mM) or ascorbate (0.25 mM) and of 1 mM 2,2′-bipyridine. The concentration of the reductively mobilized iron cations from ferritin was measured by following the absorption of the Fe(II)–bipyridine complex at 530 nm (ε = 8650 M^−1^ cm^−1^).

### Iron(III) mobilization from H− and HoS− ferritin

Iron loaded H- and HoS- ferritin (0.4 μM) have been incubated up to 3 h at 25° C in different buffers (Hepes/NaCl pH 7.4; 0.1 M acetate buffer pH 5) in the presence of 0.25 mM xylenol orange (Sigma) under aerobic conditions. At different time intervals the solutions have been centrifuged by vivaspin filters (50000 MW cut−off) (Sartorius) and 100 μl of the filtered solutions were taken to measure Fe(III) concentration after adding 700 μl of xylenol orange 0.25 mM in 20 mM H_2_SO_4_. The 100 μl taken were renewed at each time interval. The concentration of Fe(III) released from ferritin was measured by following the absorption of the Fe(III)–xylenol orange complex at 560 nm (ε = 1647 M^−1^ cm^−1^).

### Transmission electron microscopy (TEM) studies

TEM analysis of H−350, HoS-350 and HoS−1000 samples (10μM in Hepes/NaCl pH 7.4) was carried out using a JEOL JEM-3010, a 300 kV ultrahigh resolution analytical Theoretical resolution of 0.17 nm. Electron gun: Cool beam LaB6 Equipped with a Gatan US1000 CCD camera.

TEM analysis of HeLa cells was carried out after their incubation for 3 h at 37° C with H-350 1 μM or HoS-1000 2.5 μM. Control cells were incubated under the same conditions without ferritin addition. Cells were fixed with 2.5 % glutaraldehyde solution in 0.1 M phosphate buffer pH 7.4 and post-fixed in osmium tetroxide solution. Samples were then dehydrated in graded alcohol and embedded under vacuum in Epson 812. 70 nm slices were sequentially stained with 0.04% bismuth subnitrate, saturated uranyl acetate and 0.25% lead citrate and viewed with a Jeol 1400 microscopy (USA). The latter staining procedure enhances ferritin detection [36].

### Measurement of reactive oxygen species (ROS) production

Intracellular ROS production was detected using the non-fluorescent cell permeating compound, 2’,7’-dichlorodihydro-fluorescein diacetate (DCF-DA). In order to evaluate the effect of H- and HoS-ferritin on the ROS production, Hela cells were seeded at a density of 1 × 10^5^ cells in 3.5 cm—diameter dishes and were placed for 24 h in a wet (37° C) 5% CO_2_ air atmosphere incubator. Iron-loaded H- and HoS-ferritins and H- and HoS-apoferritins were incubated at protein concentration of 5 μM for 24 h at 37° C, 5% CO_2_. After the incubation, cells were washed two times with PBS and incubated with 5 μM of DCF-DA for 30’ at 37° C, 5% CO_2_ in pre-warmed EBSS buffer. Cells were then washed two times with PBS and detached using a cell scraper in 100ul of PBS. Cells were sonicated in ice for 5 sec at 30% power and the protein content of cells were determined by the commercial Bradford method. The fluorescence of cells lysates was measured using a fluorometer microplate reader Promega Glomax-multi detection system (Promega Corporation, 2800 Woods Hollow Road Madison, WI 53711 USA) using a blue module (ex 490 nm, em 510-570 nm) on samples containing 10 μg of cell proteins in 200 μl of PBS. The fluorescence intensity of iron loaded H− and HoS− ferritins and H− and HoS− apoferritins cells lysates samples was calculated as a percentage with respect to the fluorescence intensity values of untreated cells used as a control.

## SUPPLEMENTARY MATERIALS FIGURES


