# Supplementary material for: Cancer cell death induced by ferritins and the peculiar role of their labile iron pool
Source: Oncotarget. 2018 Jun 15;9(46):27974–84. doi: 10.18632/oncotarget.25416 (PMC6021343; doi:10.18632/oncotarget.25416)
Supplement: Supplementary file 1 [file oncotarget-09-27974-s001.pdf]

Cancer cell death induced by ferritins and the peculiar role of their labile iron pool

SUPPLEMENTARY MATERIALS

MATERIALS AND METHODS

Labeling of H- or HoS-apoferritin with Rhodamine B

H- or HoS-apoferritin solutions (2 mg/ml) were prepared in 0.1 M sodium carbonate buffer, pH 9. Rhodamine isothiocyanate (Rhod) (Sigma Aldrich) was dissolved in anhydrous DMSO at 1 mg/ml. For 1 mg of protein, 50 µl of Rhod solution were added very slowly in 5 µl aliquotes under stirring. Then the reaction mixture was incubated in the dark 16 h at 4° C. Then, NH_4Cl was added to the reaction to a final concentration of 50 mM and stirred for 2 h at 4° C. The unbound Rhod was separated from the conjugate by dialysis in PBS pH 7.4 using a 14000 Da cut-off membrane. Finally, the concentration of Rhod and protein were measured by a fluorometer FluoroMax 4 spectrofluorometer (Horiba Jobin Yvon, Edison, USA) and a spectrophotometer (6715 UV/Vis. Spectrophotometer Jenway, Essex, UK) with Bradford method, respectively.

Laser scan microscopy studies

Co-localization determination among H/HoS-apoferritins and lysosomes were carried on as follows. Cells were seeded in a Ibidi at a density of 2×10^4 cells/well and incubated at 37° C for 24 h, in order to allow them to adhere to the slide surface. Incubation of cells with rhodamine-labeled H or HoS-apoferritin (20 nM) was performed for 3 h. At the end of the incubation cells were washed three times with PBS and fixed in cold methanol at 4° C for ten minutes. Subsequently, cells were rinsed twice with PBS for ten minutes. In order to block the non-specific binding sites, cells were treated with 5% normal goat serum during thirty minutes. Cells were incubated overnight at 4° C with a mouse monoclonal IgG_1 anti-human Lamp-1 (H4A3 from Santa Cruz Biotechnology Inc, Dallas, Texas, USA). After washing twice in PBS ten minutes, cells were incubated with a goat polyclonal IgG (H+L) fluorescein labeled anti-mouse antibody one hour at room temperature (Molecular Probe, Eugene, Oregon USA). Finally, cells were washed with PBS three times for ten minutes and air dried. Nuclei were counterstained with DAPI. Coverslips were mounted with a glycerol/water solution (1/1, v/v). Observations were conducted under a confocal microscopy (Leica TCS SP5 imaging system).

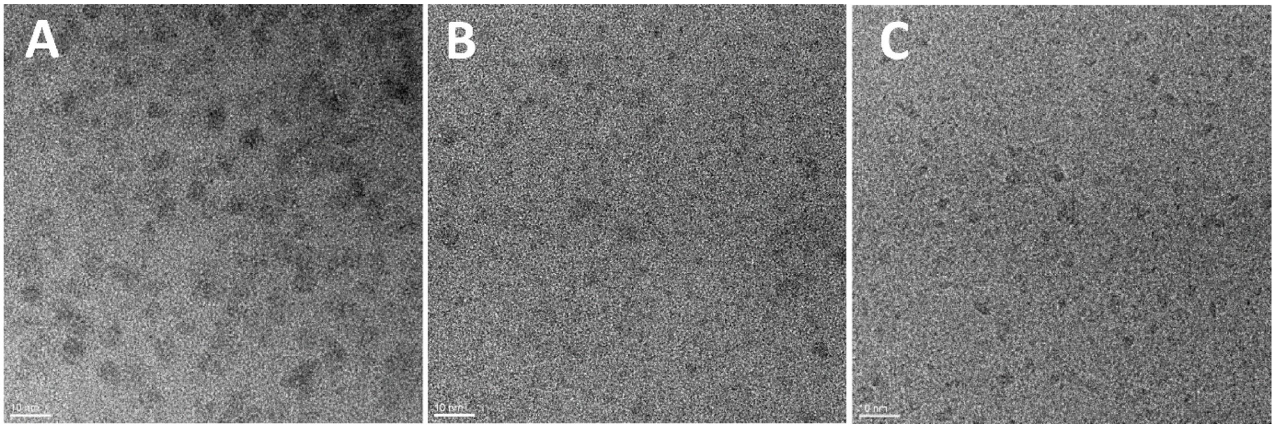

Supplementary Figure 1: TEM pictures of HoS-1000 (**A**), H-350 (**B**) and HoS-350 (C). (x200000).

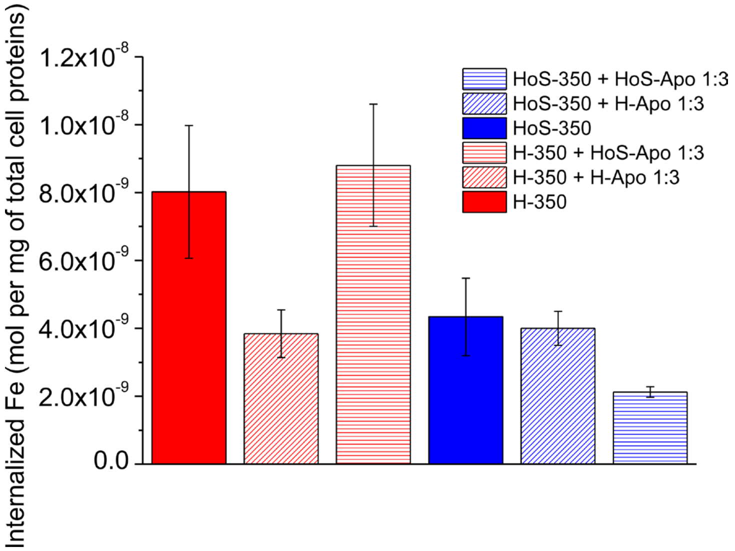

Supplementary Figure 2: Competition assay: iron uptake by Hela cells after 5 h incubation in the presence of H-350 (0.9 µM) and HoS-350 (0.9 µM) with and without the addition of an excess of H-Apo (2.7 µM) and HoS-Apo (2.7 µM). A significant decrease (>50%) of the internalized iron was observed only in the presence of the cor-responding H-Apo or HoS-Apo forms. Vice versa no effect has been detected incubating H-350 and HoS-350 with an excess of HoS-Apo and H-350, respectively.

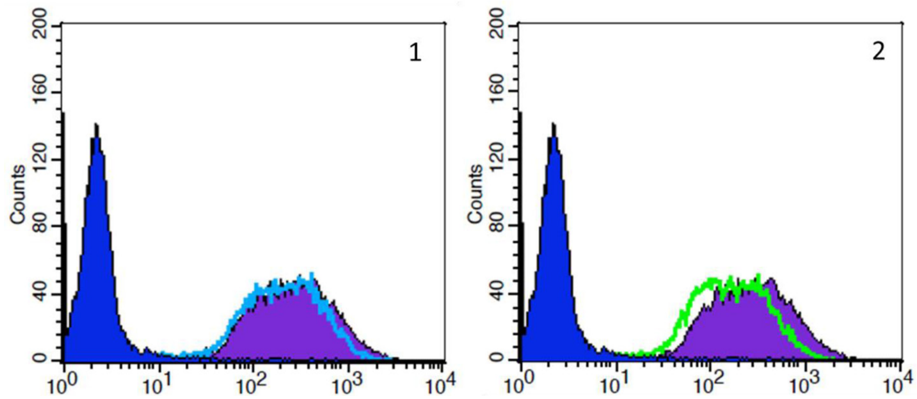

Supplementary Figure 3: FACS analysis of the TfR1 expression density on Hela cells treated for 24 h in the presence of 1) H-50 1 µM (light blue line), 2) H-350 1 µM (green line). Their PE fluorescence was analyzed using the CELLQUEST PRO program: the mean fluorescence intensity of the treated samples has been calculated as a percentage with respect to the non-treated Hela cells (violet). An isotype control antibody PE conjugated was incubated to a non-treated sample of Hela cells (blue) as negative control.

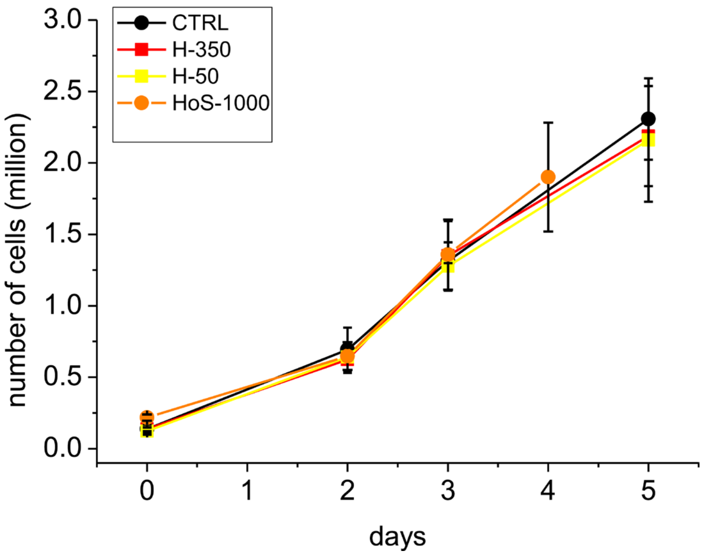

Supplementary Figure 4: Proliferation curves of Hela cells after 24 h incubation in the presence of HoS-1000 (3 µM), H-350 (0.5 µM) and H-50 (0.5 µM); negative control cells (CTRL) were incubated with the same amount of vehicle only.

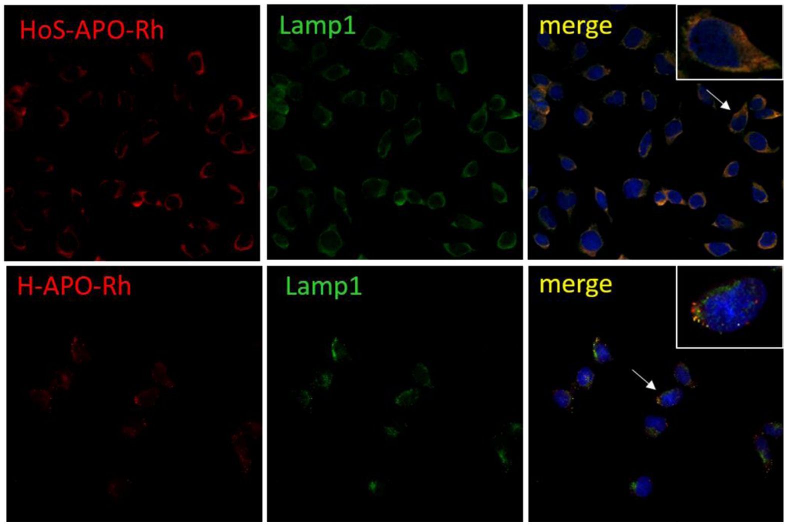

Supplementary Figure 5: Confocal Microscopy Representative images of HeLa cells incubated for 3 h with H- or HoS-APO-Rhod (red) and stained with an anti-LAMP-1 antibody (green). Nuclei were counterstained with DAPI (blue). (magnification x630) Arrows indicates the enlarged cells in the upper right panel.
